# *RtHSFA9s* of *Rhodomyrtus tomentosa* Positively Regulate Thermotolerance by Transcriptionally Activating *RtHSFA2s* and *RtHSPs*

**DOI:** 10.3390/life14121591

**Published:** 2024-12-02

**Authors:** Huiguang Li, Ling Yang, Yujie Fang, Gui Wang, Tingting Liu

**Affiliations:** 1 Key Laboratory of South China Agricultural Plant Molecular Analysis and Genetic Improvement & Guangdong Provincial Key Laboratory of Applied Botany, South China Botanical Garden, Chinese Academy of Sciences, Guangzhou 510650, China; 2 College of Life Sciences, Gannan Normal University, Ganzhou 341000, China; 3 University of Chinese Academy of Sciences, Beijing 100049, China

**Keywords:** heat shock transcription factor, thermal adaption, functional divergence, transient expression, transactivation

## Abstract

Heat shock transcription factors (HSFs) are crucial components in heat stress response. However, the contribution of the HSFs governing the inherent thermotolerance in *Rhodomyrtus tomentosa* has barely been investigated. We here compared the roles of *RtHSFA9a*, *RtHSFA9b*, and *RtHSFA9c* in heat stress tolerance. These three genes are the results of gene duplication events, but there exist vast variations in their amino acid sequences. They are all localized to the nucleus. *Arabidopsis thaliana* plants with overexpressed *RtHSFA9a* and *RtHSFA9c* outperformed the wild-type plants, while the over-accumulation of *RtHSFA9b* had little impact on plant thermotolerance. By transiently overexpressing *RtHSFA9a*, *RtHSFA9b*, and *RtHSFA9c* in *R. tomentosa* seedlings, the mRNA abundance of heat shock response genes, including *RtHSFA2a*, *RtHSFA2b*, *RtHSP17.4*, *RtHSP21.8*, *RtHSP26.5*, and *RtHSP70*, were upregulated. Transactivation assays confirmed that there exist regulatory divergences among these three genes, viz., *RtHSFA9a* has the highest transcription activity in regulating *RtHSFA2a*, *RtHSFA2b*, *RtHSP21.8*, and *RtHSP70*; *RtHSFA9c* can transcriptionally activate *RtHSFA2b*, *RtHSP21.8*, and *RtHSP70*; *RtHSFA9b* makes limited contributions to the accumulation of *RtHSFA2b*, *RtHSP21.8*, and *RtHSP70*. Our results indicate that the *RtHSFA9* genes make crucial contributions to the thermal adaption of *R. tomentosa* by positively regulating the *RtHSFA2a*, *RtHSFA2b*, and *RtHSP* genes, which provides novel insights into the *RtHSFA9* subfamily.

## 1. Introduction

Global warming driven by undue anthropogenic carbon emissions has made numerous detrimental impacts on plants. Elevated temperature exceeding the optimum constrains plant functions by affecting seed germination, photosynthetic capacity, cell growth and division, flowering, and pollen viability, resulting in plant productivity retardance [1,2,3]. Agricultural ecosystems are vulnerable to the changing climate. As estimated, extreme heat has led to a 9.1% national production deficit [4], and these impacts could be amplified by coincident stress factors such as drought and salinity [5,6]. In forestry, climate change affects forest yields and health in the aspects of physiological processes such as photosynthesis or water transport or by promoting direct or indirect drivers of mortality [7,8,9,10]. Weather pattern alterations induced by climate change, such as severe heatwaves, megadroughts, floods, heavy rain, and storms, are destroying agricultural and forestry productivity on a larger scale, raising concerns about food and forestry security [11,12,13,14]. Expanding forest coverage and improving the carbon sequestration capacity of forest ecosystems would have a significant impact on global-scale climate mitigation at the current stage [15,16]. *Rhodomyrtus tomentosa* (Ait.) Hassk is an evergreen shrub of the Myrtaceae family. It thrives in the hilly areas of Southern China, Philippines, India, Malaysia, Thailand, and Indonesia [17]. The environment where *R. tomentosa* settles always combines acidic soil, barren land, high radiation, and ambient temperature, making *R. tomentosa* a superb pioneer tree species for afforestation or rehabilitation in waste mountainous areas in tropical and subtropical regions.

Plants respond to fluctuations in circumambient temperature with widely divergent physiological and developmental modifications [18,19,20]. Elevated temperature that is above the optimum growth temperature triggers morphological and physiological alterations such as promoting stomatal opening [21,22] or initiating thermomorphogenesis to maintain growth and production [5,23]. Under noxious heat stress, the structure and function of thermo-labile macromolecules are altered, resulting in heat-aggregated proteins that are deprived of their dedicated biological activities as well as loss of plasma membrane integrity [5,18,24]. These disturbed cell structures and metabolism trigger an upsurge in reactive oxygen species (ROS), which could damage cellular components such as lipids in membranes and DNA molecules [25,26]. As the temperature continues to rise or lasts for a long period, plants can activate programmed cell death that might result in the shedding of leaves, flowers, and fruits and even the mortality of the entire plant [24,27,28]. Plants employ a set of emergency strategies to survive at this high ambient temperature, including activating molecular chaperones like heat shock proteins (HSPs) within minutes to prevent and revert protein aggregation, accumulating organic compounds, and scavenging ROS [18,20,29].

In plants, the heat shock response (HSR) and some developmental and metabolism modifications are mainly regulated by the heat shock transcription factor (HSF) family. HSFs serve as the terminal components of signal transduction [30,31]. Compared with yeast and *Drosophila*, large HSF gene families with 18–52 members have been identified in plants, which are categorized into classes A, B, and C according to the structural features of their oligomeric domains [30,32]. Among all these isoforms, the *HSFA1* genes are widely accepted to function as master activators of heat shock response (HSR) genes, thus potently mediating plant thermotolerance and thermomorphogenesis [20,33,34,35]. *HSFA2* is one of the crucial targets of *HSFA1* that greatly amplifies the activation effect of *HSFA1* under heat stress, thereby playing a vital role in modulating the transcription of HSR genes [18,36,37,38]. A recent study revealed that *HSFA2* is directly regulated by the temperature sensor *THERMO-WITH ABA-RESPONSE 1* (*TWA1*) and its partner *JASMONATE-ASSOCIATED MYC-LIKE 2* (*JAM2*) [39]. HSFA3 binds HSFA2, forms heteromeric complexes with additional HSFs, and efficiently promotes heat stress memory by positively influencing histone H3 lysine 4 (H3K4) hyper-methylation [40]. Other members of the class A family, including *HSFA4*, *HSFA6*, and *HSFA7*, are indispensable in plant heat stress response [41,42,43,44,45,46]. The biological functions of classes HSFB and HSFC have not been adequately clarified in plants. In *Arabidopsis*, *AtHSFB1* and *AtHSFB2b* negatively regulate thermotolerance, but they are essential for acquired heat tolerance [47]. Maize *ZmHSFB2b* is upregulated by heat stress and reduces thermotolerance in *Arabidopsis* and rice by affecting the expression of oxidative stress-related genes [48]. In grapevine, however, *HSFB1* positively regulates heat tolerance [31]. *LlHSFC2* coordinates with *HSFAs* to exaggerate their transactivation ability and confers thermotolerance to plants [49].

Currently, some functions of *HSFA9* have been revealed in several plants, such as *Arabidopsis*, sunflower (*Helianthus annuus*), grape (*Vitis vinifera*), and *Medicago truncatula*. *HSFA9* belongs to class HSFA and is thought to be seed-specific [50,51], whilst current evidence reveals that *HSFA9* acts as an important hub in mediating embryogenesis, germination, photomorphogenesis, and stress protection. Overexpressing grape *VvHSFA9* in *Arabidopsis* plants enhances seed germination under unstressed conditions and late-flowering phenotypes [52]. *HSFA9* contributes to seed maturation and longevity by potently activating HSP genes in *Arabidopsis* [51], sunflower [53,54], and *M. truncatula* [55]. These are related to the ABA signaling pathway [50,51], and overexpressing *HSFA9* enhances severe seed dehydration and oxidative stress tolerances in sunflower [54,56] and *M. truncatula* [55]. In the seedling stage, *HaHSFA9* regulates plant morphogenesis by promoting the transcription of genes regarding photomorphogenesis or photoreceptors [57,58]. *HSFA9* also participates in light protection. Ectopic expressing *AtHSFA9* in tobacco protects photosynthetic membranes [59]; sunflower HaHSFA9 interacts with tobacco UV RESISTANCE LOCUS 8 (NtUVR8) and maintains its nuclear localization, which in turn enhances UV-B responses and photoprotection in transgenic tobacco [60]. In addition, AtHSFA9 can interact with AtHSFA2 in *Arabidopsis*, and in sunflower, HaHSFA9 functionally interacts with DROUGHT-RESPONSIVE ELEMENT-BINDING FACTOR 2 (HaDREB2) [53,61], uncovering the role that HSFA9 plays in plant thermotolerance.

We previously identified the HSF family of *R. tomentosa* and illustrated a basic thermal adaption mechanism mediated by *RtHSFA2a* and *RtHSFA2b* [62]. We have noticed that subclass HSFA9 has been enlarged in *R. tomentosa* (3 members) and *Eucalyptus grandis* (16 members), indicating a special genetic basis in regulating plant development and stress responses. In this study, we accessed the functional effects of the *RtHSFA9* genes on the heat stress response and disclosed their regulatory networks in *R. tomentosa*. Our results demonstrate that *RtHSFA9s* play roles in plant thermotolerance; further, they have different transcription activities in regulating heat stress response genes.

## 2. Materials and Methods

### 2.1. Plant Materials and Growth Conditions

Seeds of *R. tomentosa* were soaked with 400 mg/L gibberellin A3 (GA3; purchased from Biorigin, Beijing, China) for two days and sown in soil. They were kept at 25 °C supplied with a 16/8 (light/dark) photoperiod and 120 μmol m^−2^s^−1^ light intensity. Seeds were collected from the South China National Botanical Garden (Guangzhou, Guangdong Province, China). The pots were irrigated according to the moisture level of the soil. Germinated seedlings with two true leaves were used for gene transformation.

We used the *Arabidopsis thaliana* Columbia-0 (Col-0) ecotype in this study. Seeds of Col-0 were sterilized directly with 75% ethanol (purchased from GHTECH, Guangdong Province, China) for 15 min; then, they were rinsed with 100% ethanol two times and exposed to air on sterile filter paper. After ethanol was volatilized, seeds were sown on half-strength Murashige and Skoog medium (1/2 MS; purchased from Coolaber Science & Technology, Beijing, China) containing 0.54% agar (purchased from Coolaber Science & Technology) and 1% sucrose (purchased from Guangzhou Chemical Reagent Factory, Guangdong Province, China). They were stratified at 4 °C for two days in darkness and then transferred into a phytotron supplied with a photoperiod of 16/8 (light/dark) at 22 °C. Six days after that, seedlings of *Arabidopsis* were transferred into the soil and grown under the same conditions.

### 2.2. Phylogenetic Analysis and Sequence Alignment

The amino acid sequences of the RtHSFA9a, RtHSFA9b, and RtHSFA9c were used to identify their homologous genes in the genomes of *A. thaliana*, *E. grandis*, *Populus trichocarpa*, *Prunus persica*, *Glycine max*, *Salix purpurea*, *Citrus sinensis*, *Manihot esculenta*, *Trifolium pratense*, *Linum usitatissimum*, *Fragaria vesca*, *Malus domestica*, *Betula platyphylla*, and *Ricinus communis* in Phytozome (version 13; https://phytozome-next.jgi.doe.gov/; last accessed in 30 August 2024). These sequences were aligned by MAFFT software (version 7; https://mafft.cbrc.jp/alignment/server/index.html; last accessed on 30 August 2024); then, RAxML-NG was used to construct a phylogenetic tree by the maximum likelihood (ML) method with a bootstrap value of 1000 [63]. The output of RAxML-NG was visualized by the R package GGTREE [64].

The protein sequences of RtHSFA9a, RtHSFA9b, RtHSFA9c, and AtHSFA9 were used for multiple-sequence comparison by using Jalview software (version 2.11.3.3; https://www.jalview.org/; last accessed on 30 August 2024), and the conserved domains were predicted by SMART (http://smart.embl-heidelberg.de/; last accessed on 30 August 2024) with default parameters.

### 2.3. Gene Cloning, Plasmid Construction, and Generation of Transgenic Arabidopsis Plants

The coding sequences (CDSs) of *RtHSFA9a*, *RtHSFA9b*, and *RtHSFA9c* were amplified by using the sequence-specific primers (Table S1) from the complementary DNA (cDNA) of *R. tomentosa* prepared previously [62]. To construct the overexpression vectors, they were inserted into a modified pCAMBIA1300 binary vector separately under the control of the promoter of *AtUBQ10* (pAtUBQ10). They were then introduced into *Agrobacterium tumefaciens* (strain GV3101) to transform *A. thaliana* by using the floral dip method [65]. T3 homozygous lines overexpressing *RtHSFA9a*, *RtHSFA9b*, and *RtHSFA9c* (hereafter denoted by OEHSFA9a, OEHSFA9b, and OEHSFA9c, separately) were used for further experiments.

### 2.4. Subcellular Localization

To visualize the subcellular localization of the *RtHSFA9* genes, the CDSs of *RtHSFA9a*, *RtHSFA9b*, and *RtHSFA9c* were fused with the GFP reporter separately. The histone *H2B* of *Arabidopsis* (*AtH2B*) was fused with mCherry and used to indicate the nucleus. These cassettes were cloned into a modified pCAMBIA1300 binary vector separately, driven by pAtUBQ10. These reconstructed vectors were then introduced into *A. tumefaciens* (strain GV3101) and used for tobacco (*Nicotiana benthamiana*) leaf infiltration. The infected tobacco plants were kept in darkness for 48 h. A laser confocal fluorescence microscope (Leica TCS SP8 STED 3X; Leica, Wetzlar, Germany) was then used to detect the fluorescence signals (excitation at 488 nm and emission at 505–540 nm for GFP; excitation at 594 nm and emission at 598–684 nm for mCherry) [36].

### 2.5. Heat Stress Treatment of Transgenic Arabidopsis

Three-day-old seedlings of Col-0, OEHSFA9a, OEHSFA9b, and OEHSFA9c germinated on 1/2 MS medium were transferred into new plates. After being grown under normal conditions for another 3 days, the plants were suddenly subjected to 45 °C for 135 min and then recovered at 22 °C for 7 days. Photographs were taken after that, and the survival rate and the total chlorophyll content were analyzed as described before [62].

### 2.6. Transient Transformation of R. tomentosa, RNA Extraction, and Reverse Transcription Quantitative PCR (RT-qPCR) Analysis

The overexpression vectors of *RtHSFA9a*, *RtHSFA9b*, and *RtHSFA9c*, as well as the empty vector, were transiently transformed into *R. tomentosa* seedlings as we described previously [62]. Three days after transformation, leaves of seedlings transformed with the empty vector (denoted by VT), *RtHSFA9a* (denoted by OxHSFA9a), *RtHSFA9b* (denoted by OxHSFA9b), and *RtHSFA9c* (denoted by OxHSFA9c) were collected separately and frozen in liquid nitrogen immediately and used for RNA extraction.

Samples were ground into powders in liquid nitrogen, and the cetyltrimethylammonium bromide (CTAB) method was used to extract total RNA according to the procedure described previously [66]. CTAB was purchased from Macklin (Shanghai, China). The cDNA was synthesized by a HiScript II 1st Strand cDNA Synthesis Kit (Vazyme, Nanjing, China) following the manufacturer’s guidelines. RT-qPCR was performed on a Quantagene q225 real-time PCR system (Kubo Technology, Beijing, China) by using the ChamQ SYBR qPCR Master Mix by Vazyme, and the primers used for Rt-qPCR are listed in Table S1. Each sample contained three duplicates. In *R. tomentosa*, the relative mRNA abundance rates of genes were normalized by the 2^−ΔΔCT^ method [67] according to the expression level of *R. tomentosa Actin* (*RtActin*) in *R. tomentosa* [68] or *AtActin2* in *Arabidopsis* [69,70].

### 2.7. Transactivation Activity Assays in Tobacco Leaves

For transcription activity analysis, the promoters of putative target genes of *RtHSFA9s* (around 2 kb upstream of the translation initiation site) were cloned from the genome DNA of *R. tomentosa* and then inserted into the double reporter vector pGreenII-0800-LUC to drive the Firefly luciferase (LUC) gene. The Renilla luciferase (REN) was driven by the CaMV35s promoter in the same vector and used as the internal control. These reconstructed vectors were used as reporters. The overexpression vectors of *RtHSFA9s* and the empty vector of the modified pCAMBIA1300 were used as effectors. All these vectors were introduced into *A. tumefaciens* (strain GV3101) and then co-infiltrated into the *N. benthamiana* leaves. Firefly luciferase activity was observed by a Nightshade evo LB985 In Vivo Plant Imaging System (Berthold Technologies, Bad Wildbad, Germany), and LUC and REN activities were determined by using the Dual-Luciferase Reporter Assay Kit by Vazyme according to the manufacturer’s instructions. The transactivation activities of *RtHSFA9s* to their targets were indicated by the ratio of LUC to REN. Three duplicates were performed in each sample.

### 2.8. Statistical Analysis

All the values were averages of at least three independent replicates, and they were presented as the mean values ± SDs. One-way ANOVA was conducted by R (version 4.4.1; https://cran.r-project.org/; last accessed on 30 August 2024), followed by post hoc Tukey’s Honestly Significant Difference (Tukey’s HSD) tests to evaluate the statistical significance in each group (*p* < 0.05). Values sharing the same lowercase letters indicated that there was no statistical difference.

## 3. Results

### 3.1. Sequence Characteristics, and Phylogenetic and Subcellular Localization Analyses of RtHSFA9a, RtHSFA9b, and RtHSFA9c

**Figure 1:**
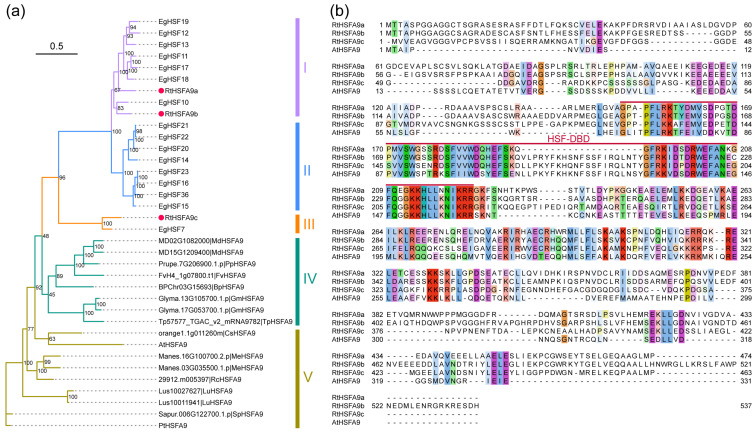
Evolutionary relationship and sequence comparison of RtHSFA9a, RtHSFA9b, and RtHSFA9c. (**a**) Phylogenetic analysis of HSFA9 genes. Homologous genes of RtHSFA9a, RtHSFA9b, and RtHSFA9c of *A. thaliana*, *E. grandis*, *P. trichocarpa*, *P. persica*, *G. max*, *S. purpurea*, *C. sinensis*, *M. esculenta*, *T. pratense*, *L. usitatissimum*, *F. vesca*, *M. domestica*, *B. platyphylla*, and *R. communis* were derived from Phytozome and used for phylogenetic tree construction. The maximal likelihood tree was generated by RAxML-NG with a bootstrap value of 1000. The number on the branch indicates the percentages of trees in which the associated taxa clustered together in the bootstrap analysis. (**b**) Multiple-sequence alignment of RtHSFA9a, RtHSFA9b, RtHSFA9c, and AtHSFA9. The conserved HSF-type DNA-binding domain (HSF-DBD) is indicated by the red line.

**Figure 2:**
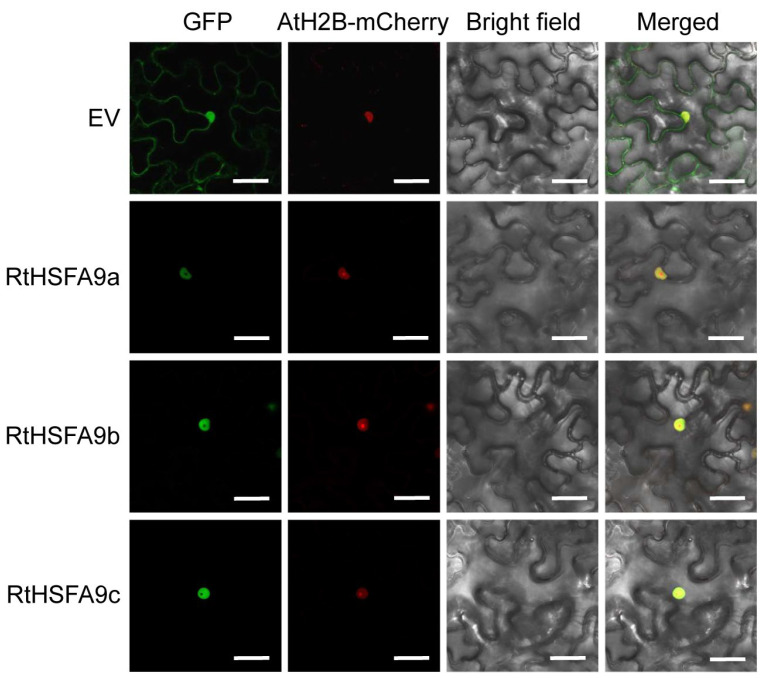
Subcellular localization of RtHSFA9s. *RtHSFA9a*, *RtHSFA9b*, and *RtHSFA9c* were fused with GFP, and a nuclear marker gene, *AtH2B*, was fused with mCherry. They were all driven by pAtUBQ10 and transfected into *N. benthamiana* leaves. EV, empty vector that contained pAtUBQ10::GFP. EV was used as control. Bar = 10 μm.

The coding sequences of *RtHSFA9a*, *RtHSFA9b*, and *RtHSFA9c* were obtained from the cDNA of *R. tomentosa*. Sequence analysis revealed that they are 1488 bp, 1527 bp, and 1392 bp in length, predicted to encode 495, 508, and 463 amino acids, respectively, and contain a putative HSF-binding domain. The molecular weights of RtHSFA9a, RtHSFA9b, and RtHSFA9c were around 55.13 KD, 56.57 KD, and 51.00 KD, with isoelectric points of 5.3, 5.37, and 5.02, respectively. The amino acid sequences varied among the three RtHSFA9s, and they shared limited identities with AtHSFA9 except for the HSF-type DNA-binding domain (HSF-DBD) (Figure 1b). The results of the phylogenetic analysis in various plants reveal that these HSFA9 amino acid sequences can be classified into five clades, and the RtHSFA9s has the closest evolutionary relationships with those of *E. grandis*; nevertheless, RtHSFA9a shares higher sequence identity with RtHSFA9b than RtHSFA9c (Figure 1a). The results of the subcellular localization assay indicate that RtHSFA9a, RtHSFA9b, and RtHSFA9c are all localized to the nucleus (Figure 2).

### 3.2. Effects of RtHSFA9s on Heat Stress Tolerance in Arabidopsis

**Figure 3:**
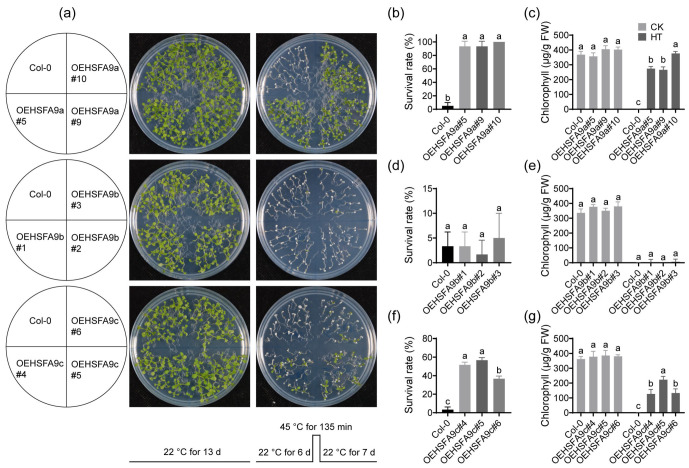
Effects of acute heat stress on *Arabidopsis* seedlings with overexpressed *RtHSFA9s*. (**a**) Performance of Col-0 and *RtHSFA9s*-overexpressing plants under normal and heat stress conditions. Col-0 and transgenic plants grown at 22 °C for 6 days were suddenly exposed to 45 °C for 135 min and then transferred back to 22 °C for recovery. Photographs were taken after 7 days of recovery. Plants grown at 22 °C for 13 days were used as control. Comparable results were obtained after that. (**b**) The survival rate of Col-0 and *RtHSFA9a*-overexpressing plants after heat stress. (**c**) Total chlorophyll content in Col-0 and *RtHSFA9a*-overexpressing plants grown under normal conditions and after recovering from heat stress. (**d**) The survival rate of Col-0 and *RtHSFA9b*-overexpressing plants after heat stress. (**e**) Total chlorophyll content in Col-0 and *RtHSFA9b*-overexpressing plants grown under normal conditions and after recovering from heat stress. (**f**) The survival rate of Col-0 and *RtHSFA9c*-overexpression plants after heat stress. (**g**) Total chlorophyll content of Col-0 and *RtHSFA9c*-overexpressing plants grown under normal conditions and after recovering from heat stress. Data shown are means ± SDs (*n* = 3). The significances among different lines under normal or heat stress conditions were evaluated by one-way ANOVA followed by post hoc Tukey’s HSD (*p* < 0.05). Samples sharing the same letters showed no significant difference.

*HSFA9* genes play positive roles in regulating the plant heat stress response in *Arabidopsis* [61] and sunflower [53]. We previously identified that *RtHSFA9a* and *RtHSFA9c* derive from *RtHSFA2a* and *RtHSFA2b* in *R. tomentosa* [62]. To investigate the functions of *RtHSFA9s*, we generated transgenic *Arabidopsis* plants with overexpressed *RtHSFA9a*, *RtHSFA9b*, and *RtHSFA9c* separately (denoted by OEHSFA9a, OEHSFA9b, and OEHSFA9c, respectively). The lines with comparable mRNA abundance of *RtHSFA9a*, *RtHSFA9b*, or *RtHSFA9c* (Figure S1) were used to evaluate the performance under sudden heat stress. As shown in Figure 3a, OEHSFA9a, OEHSFA9b, and OEHSFA9c plants exhibited similar appearances with wild-type *Arabidopsis* plants (Col-0) under normal conditions. However, most of Col-0 and OEHSFA9b plants did not survive after having been subjected to 45 °C for 135 min and recovered at 25 °C for 7 days, while over 93% of OEHSFA9a plants and around 36.7~56.7% of OEHSFA9c plants survived after heat stress (Figure 3a,b,d,f). Statistical differences among OEHSFA9a, OEHSFA9b, OEHSFA9c, and Col-0 plants were underscored by evaluating total chlorophyll content. These plants showed similar total chlorophyll contents in the control groups (Figure 3c,e,g), whereas after heat stress treatment, total chlorophyll contents in OEHSFA9a and OEHSFA9c plants were significantly higher than that in Col-0 plants, although they were lower than the control group (Figure 3c,g). Nevertheless, the performance of OEHSFA9b was similar to Col-0 (Figure 3e). All these manifestations disclosed a forceful protective role of *RtHSFA9a* and *RtHSFA9c* against heat stress, while *RtHSFA9b* plays neutral roles in thermotolerance in *Arabidopsis*.

### 3.3. Regulatory Networks of RtHSFA9s in Heat Stress Response in R. tomentosa

**Figure 4:**
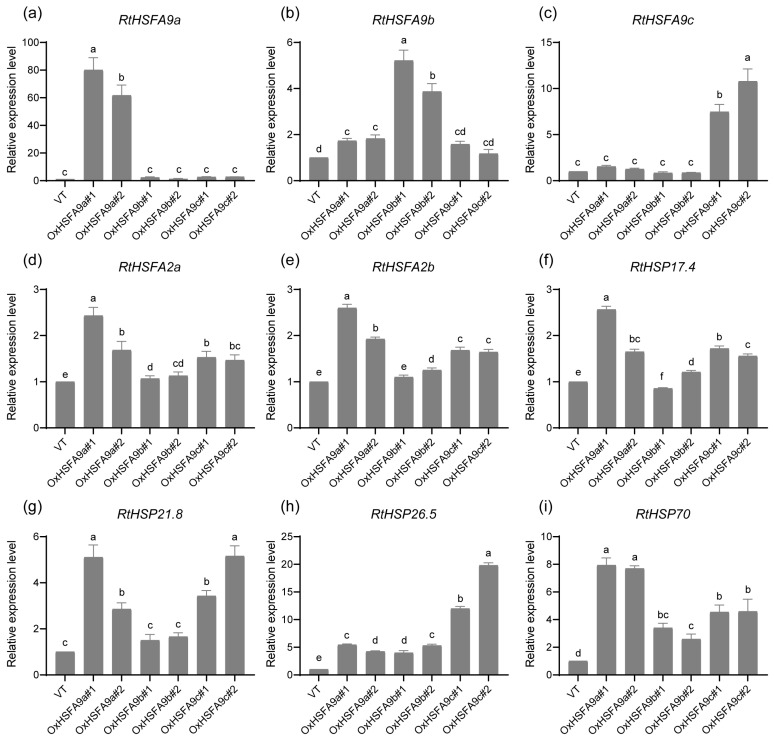
Endogenous heat stress response gene expression of empty vector (VT) and *RtHSFA9s*-overexpressing seedlings of *R. tomentosa*. *RtHSFA9a*, *RtHSFA9b*, *RtHSFA9c*, and empty vector (VT) were transiently expressed in one-month-old *R. tomentosa* seedlings, and RT-qPCR was performed with specific primers (Table S1). The VT line was used as a control. (**a**–**c**) Verification of mRNA abundance of *RtHSFA9a*, *RtHSFA9b*, and *RtHSFA9c* in OxHSFA9a, OxHSFA9b, OxHSFA9c, and VT lines. (**d**–**i**) Quantification of heat stress response genes in transient expression seedlings of *R. tomentosa*. Data shown are means ± SDs (*n* = 3). The significances among different lines were evaluated by one-way ANOVA followed by post hoc Tukey’s HSD (*p* < 0.05). Samples sharing the same letters showed no significant difference.

*HSFA9* regulates the expression of heat stress response genes in *Arabidopsis* [51,61]. To explore the regulatory network of *RtHSFA9s* in *R. tomentosa*, we transiently expressed *RtHSFA9a*, *RtHSFA9b*, and *RtHSFA9c* in *R. tomentosa* seedlings, and the empty vector (VT) was used as the control. The mRNA abundance rates of putative targets were quantified by RT-qPCR. *RtHSFA9s* were successfully expressed in *R. tomentosa*, for we detected 61.7~80.0-fold overexpression of *RtHSFA9a* in the OxHSFA9a lines, 3.8~5.2-fold overexpression of *RtHSFA9b* in the OxHSFA9b lines, and 7.4~10.7-fold overexpression of *RtHSFA9c* in the OxHSFA9c lines compared with VT plants (Figure 4a–c). We then investigated the expression levels of *RtHSFA2a* and *RtHSFA2b*, two pivotal genes in regulating the heat stress response in *R. tomentosa*, in these *RtHSFA9s*-overexpressing lines. The expression levels of *RtHSFA2a* were higher in the OxHSFA9a lines than other lines, with around 1.6~2.4-fold higher levels than in VT lines; the mRNA abundance of *RtHSFA2a* in the OxHSFA9c lines was upregulated by around 1.4~1.5 times compared with VT lines, which had lower abundance than the OxHSFA9a lines but higher than the other lines; finally, in the OxHSFA9b lines, the expression levels of *RtHSFA2a* showed no perceptible differences compared with the VT lines (Figure 4d). A similar tendency could be found when detecting the expression levels of *RtHSFA2b* (Figure 4e). These results indicate that *RtHSFA9a* and *RtHSFA9c* could transcriptionally activate *RtHSFA2a* and *RtHSFA2b* in *R. tomentosa*, while *RtHSFA9b* had no discernible effects in regulating these two genes. We then detected the transcriptional levels of several heat stress response genes, including *RtHSP17.4*, *RtHSP21.8*, *RtHSP26.5*, and *RtHSP70*. The expression levels of *RtHSP17.4* and *RtHSP21.8* in OxHSFA9a and OxHSFA9c lines were remarkably higher than in the VT line, while in the OxHSFA9b lines, they showed no significant difference or were slightly higher than in the VT line (Figure 4f–g). The expression levels of *RtHSP70* were similar to *RtHSP21.8* (Figure 4i). Different from all the other target genes, the mRNA abundance rates of *RtHSP26.5* in the OxHSFA9c lines (11.9~19.8-fold higher than in the VT line) were higher than in other lines, although *RtHSP26.5* was also upregulated in the OxHSFA9a and OxHSFA9b lines (around 3.9~5.4-fold higher expression than in the VT line) (Figure 4h).

### 3.4. RtHSFA9a, RtHSFA9b, and RtHSFA9c Showed Different Transactivation Activities in Regulating Heat Stress Response Genes

**Figure 5:**
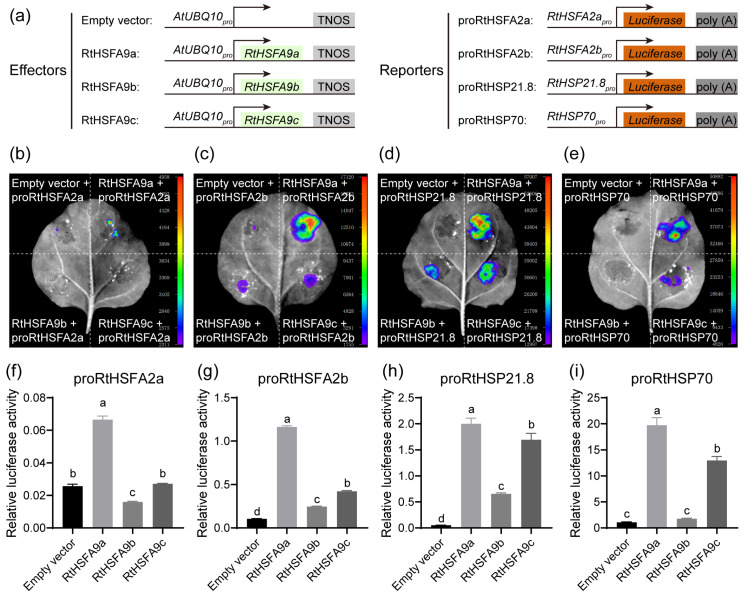
*RtHSFA9s* transcriptionally regulate heat shock response genes. (**a**) Diagrams demonstrating the effectors and reporters used for transactivation assays in *N. benthamiana* leaves. The CDSs of the *RtHSFA9s* genes were driven by pAtUBQ10 and used as effectors, and the empty vector was used as a control. Promoter regions (around 2 kb upstream the of translation initiation site) of *RtHSFA2a*, *RtHSFA2b*, *RtHSP21.8*, and *RtHSP70* were used to drive the firefly luciferase of vector pGreenII-0800-LUC and used as reporters. The Renilla luciferase gene was driven by the CaMV-35S promoter and used as the internal control. Effectors and reporters were mixed as indicated in (**b**–**e**) and infiltrated into *N. benthamiana* leaves. (**b**–**e**) Representative images of the transactivation of *RtHSFA2a*, *RtHSFA2b*, *RtHSP21.8*, and *RtHSP70* promoters by *RtHSFA9s* using luciferase activity assay. *N. benthamiana* leaves infiltrated by different combinations of effectors and reporters were observed and photographed 48 h after infection. (**f**–**i**) Relative luciferase activities in (**b**–**e**) were measured. Data shown are means ± SDs (*n* = 3). The significances among different samples were evaluated by one-way ANOVA followed by post hoc Tukey’s HSD (*p* < 0.05). Samples sharing the same letters showed no significant difference.

According to all the results presented above, we conjectured that *RtHSFA9a*, *RtHSFA9b*, and *RtHSFA9c* have different transcription activities from their targets, and *RtHSFA9a* might be more important in mediating thermotolerance in *R. tomentosa*. We amplified the promoters (around 2 kb upstream of the transcription initiation site) of *RtHSFA2a*, *RtHSFA2b*, *RtHSP21.8*, and *RtHSP70* to drive firefly luciferase and used them as reporters (Figure 5a). They were co-infiltrated into tobacco leaves with the overexpression vectors of *RtHSFA9a*, *RtHSFA9b*, and *RtHSFA9c*, as well as the empty vector as specified in Figure 5b–e. The results show that RtHSFA9a greatly activated all these targets, and its transcription activity was significantly higher than RtHSFA9b and RtHSFA9c (Figure 5b–i). RtHSFA9c could transactivate *RtHSFA2b*, *RtHSP21.8*, and *RtHSP70* but could not directly activate *RtHSFA2a* (Figure 5b–i). By comparison, RtHSFA9b slightly transrepressed *RtHSFA2a* (Figure 5b,f) while mildly transactivating *RtHSFA2b* (Figure 5c,g), and it could transactivate *RtHSP21.8* and *RtHSP70* (Figure 5d–e,h–i). These results disclose that RtHSFA9a acts as a potent activator in regulating *RtHSFA2s* along with other *RtHSPs* in *R. tomentosa* and RtHSFA9c tends to transactivate *RtHSFA2b* and *RtHSPs* in response to heat stress, while RtHSFA9b makes finite contributions to the thermotolerance of *R. tomentosa* compared with RtHSFA9a and RtHSFA9c.

## 4. Discussion

There is overwhelming evidence indicating that climate change is intensifying due to the overall effects of multiple factors, such as the expansion of the global population, the development of industrialization, and deforestation and degradation across the world [71,72,73,74]. Afforestation and restoration are crucial strategies to mitigate climate change and achieve carbon neutrality [75,76]. Flourishing in the tropical and subtropical regions, *R. tomentosa* favors sunshine and possesses many advantages a priori in enduring high temperatures and humidity, acidic soil, barren land, insect pests, and pathogen infection, making it a splendid species for afforesting difficult sites and a superb model plant for adversity [62]. Investigating the hereditary basis of *R. tomentosa* in thermal adaption could provide a novel understanding of how plants respond to high temperatures as well as gene resources and strategies for breeding heat-tolerant plants.

In model plants or major crops such as *Arabidopsis*, rice, wheat, and maize, HSFs are well known as the main hubs in regulating plant thermotolerance [33,40,44,77,78], but the functions of *HSFA9* in the heat stress response are still far from being fully understood, especially in woody plants. We previously identified three members in the HSFA9 subfamily named *RtHSFA9a*, *RtHSFA9b*, and *RtHSFA9c* in *R. tomentosa* [62]. Although they are classified into the same class, these three RtHSFA9s show large variations in amino acid sequences, and they share only 25.96%, 24.76%, and 31.33% identity rates with AtHSFA9, respectively (Figure 1b). In evolution, they are close to the HSFA9s genes of *E. grandis* whilst distant from AtHSFA9 according to the phylogenetic tree (Figure 1a). These indicate that *RtHSFA9s* might function differently from *Arabidopsis*. Genetic evidence indicates that *AtHSFA9* is specifically expressed in seeds [61,79], and seed-specific elements are present in the promoter of *AtHSFA9* [51]. By controlling a complex regulatory network, *AtHSFA9* links seed longevity, seedling photomorphogenesis, and dehydration tolerance [54,58]. *HSFA9* has similar functions in sunflower seeds. Sunflower *HaHSFA9* is specifically expressed during embryogenesis [80]. Overexpressing *HaHSFA9* in tobacco attenuates hypocotyl growth in darkness and accelerates initial photosynthetic development [57]. In *R. tomentosa*, however, the expression of *RtHSFA9s* could be detected in the root, leave, flower, and fruit in the absence of environmental stress [62,81], indicating that they might be involved in basal signaling in growth regulation or stress response.

The RtHSFA9 subfamily has enlarged during evolution, for many plants, such as *Arabidopsis*, rice, and *Populus*, contain only one HSFA9 [82,83,84]. Interestingly, the genome of *E. grandis* encodes 16 *HSFA9* genes, enlarged mainly by tandem duplications [85]. Similar to *R. tomentosa*, *Eucalypts*, as one of the most widely planted hardwood trees with splendid growth rates and superior wood properties, has remarkable adaptability in tropical-to-temperate regions [86]. Considering the absence of *HSFA9* in many genomes, such as *Sorbus pohuashanensis* [87], *sesame* [88], and *Brassica nalpus* [89], we speculate that the amplification of the HSFA9 subfamily confers additional abilities to these two species to cope with warm temperature and high humidity. Moreover, *RtHSFA9s* originate from *RtHSFA2s* as the result of whole-genome duplication. According to our previous study, *RtHSFA9a* is one of the paralogous genes of *RtHSFA2a* and *RtHSFA2b*, and *RtHSFA9b* is homologous to *RtHSFA2a* [62]. This implies that *RtHSFA9s*, especially *RtHSFA9a* and *RtHSFA9c*, might have similar regulatory networks compared with *RtHSFA2a* and *RtHSFA2b* in *R. tomentosa*, thereby acting as a positive regulator in thermal adaption.

We then assessed the basal thermotolerance of transgenic *Arabidopsis* seedlings with overexpressed *RtHSFA9a*, *RtHSFA9b*, and *RtHSFA9c*. The results show that *Arabidopsis* plants with overexpressed *RtHSFA9a* and *RtHSFA9c* outperformed wild-type plants under sudden severe heat stress, and *RtHSFA9a* had better performance compared with *RtHSFA9c* (Figure 3). Overexpressing *RtHSFA9b* showed no alteration in basal thermotolerance in *Arabidopsis* (Figure 3a). This is consistent with the results of *RtHSFA2a* and *RtHSFA2b* in *Arabidopsis*, emphasizing that *RtHSFA9a* and *RtHSFA9c* which derive from *RtHSFA2a* and/or *RtHSFA2b* play important roles in plant basal heat tolerance.

Plant HSPs, including sHSPs, HSP70, and HSP101, act as protein chaperones; they can trap partially denatured proteins on their surface and restore the refolding of denatured proteins to native physiological conditions [90,91]. HSPs are important for surviving high temperatures and other stresses [91,92,93,94]. Mounting evidence indicates that HSFA9 takes part in regulating HSPs in plants. A set of HSP genes encompassing *HSP17.4-CI* and *HSP101* require HSFA9 for full activation [51,79], and the expression levels of *HSFA2*, *HSP17.6*, and *HSP101* are impaired in *hsfa9*-mutant *Arabidopsis* [61]. In our study, the overexpression of *RtHSFA9s* resulted in constitutive exaggerated expression of HSP genes including *RtHSP17.4*, *RtHSP21.8*, *RtHSP26.5*, and *RtHSP70* in *R. tomentosa* (Figure 4f–i), and the transactivation assays showed that there exist functional divergences in RtHSFA9s in regulating these genes, for RtHSFA9a and RtHSFA9c can potently activate *RtHSP21.8* and *RtHSP70*, while RtHSFA9b has much weaker transcription activity than RtHSFA9a and RtHSFA9c in regulating these two genes (Figure 5d–e,h–i). These results are consistent with our previous conclusion expounding that *RtHSFA2s* are pivotal regulators of HSP genes in *R. tomentosa* [62].

HSFs are crucial transcription factors that regulate *HSPs* or other heat stress response genes in plants [30,70]. In the *RtHSFA9a-* and *RtHSFA9c*-overexpressing *R. tomentosa* seedlings, we noticed that the expression levels of *RtHSFA2a* and *RtHSFA2b* were upregulated (Figure 4d–e), and the direct regulatory relationships were further confirmed by transient transactivation assays in tobacco leaves (Figure 5b–c,f–g). Moreover, RtHSFA9a had much higher activity in transactivating *RtHSFA2a* and *RtHSFA2b* than RtHSFA9b and RtHSFA9c, indicating that RtHSFA9a is an influential regulator of the heat stress response in *R. tomentosa*. These results suggest the notion that the amplification of the RtHSFA9 subfamily could provide more regulatory flexibility in manipulating the expression of *RtHSFA2a*, *RtHSFA2b*, and other heat stress response genes, thereby improving heat stress tolerance in *R. tomentosa*, and *RtHSFA9a* and *RtHSFA9c* present greater potency in mediating these pathways.

**Figure 6:**
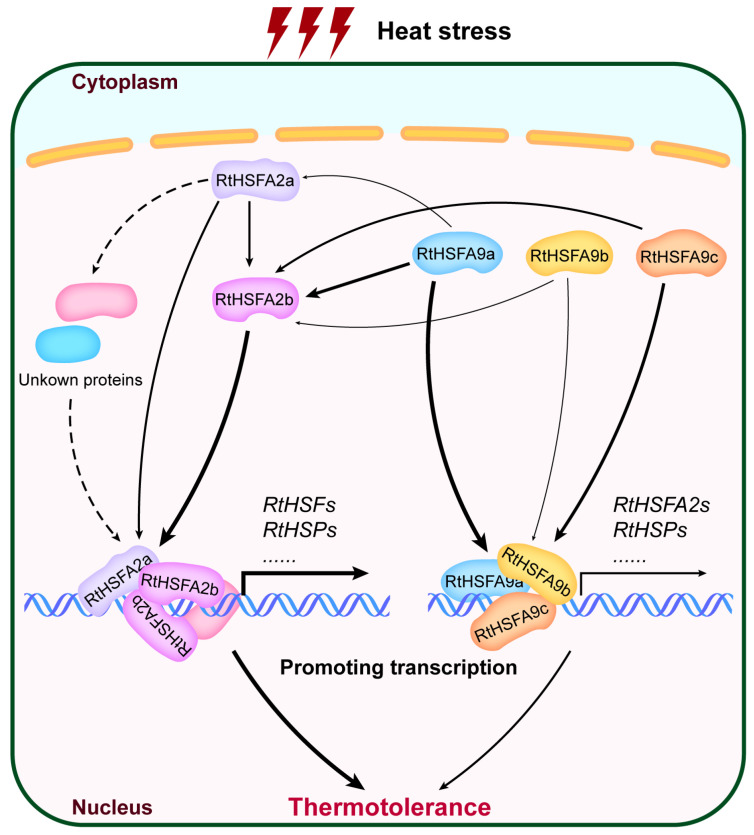
Integrated regulatory network of thermotolerance mediated by *RtHSFA2s* and *RtHSFA9s* in *R. tomentosa*. Under heat stress, *RtHSFA2b* is acutely accumulated and plays pivotal roles in plant thermotolerance, thereby dramatically transactivating the transcription of heat stress response genes such as other *RtHSFs* (heat shock transcription factors of *R. tomentosa*) and *RtHSPs* (heat shock proteins of *R. tomentosa*) in *R. tomentosa*. *RtHSFA2a* moderately responds to warm temperatures; it can transcriptionally activate *RtHSFA2b* and some *RtHSPs*, as well as certain hidden factors, to regulate plant heat stress tolerance. *RtHSFA9a* positively regulates the transcription of *RtHSFA2a*, *RtHSFA2b*, and some *RtHSPs*, including *RtHSP21.8* and *RtHSP70*, in *R. tomentosa*, thereby dramatically enhancing plant heat stress tolerance. *RtHSFA9b* and *RtHSFA9c* can activate the expression of *RtHSFA2b* and some *RtHSP* genes, consequently taking part in thermal adaption in *R. tomentosa*. In comparison with *RtHSFA9b*, *RtHSFA9c* has higher transcription activity in regulating *RtHSFA2b* and *RtHSP* genes and therefore confers promising thermotolerance to plants.

Nowadays, global warming has brought more and more natural disasters with increased magnitude, frequency, and duration. These alterations in climate have caused enormous impacts on agriculture, ecosystem stability, and human life globally [72,95,96,97,98]. Illustrating the mechanisms underlying plant adaption to warm temperatures becomes an exigent task for tree breeding. We conclude here that all three *RtHSFA9s* of *R. tomentosa* play roles in the heat stress response, but there exist functional divergences among them. *RtHSFA9a* can drastically improve plant thermotolerance by transactivating *RtHSFA2a* and *RtHSFA2b*, as well as a set of *HSPs* including *RtHSP21.8* and *RtHSP70*; *RtHSFA9c* takes part in heat stress endurance by positively activating *RtHSFA2b*, *RtHSP21.8*, and *RtHSP70*; *RtHSFA9b* weakly contributes to the increase in the levels of *RtHSFA2b*, *RtHSP21.8*, and *RtHSP70* (Figure 6). This study roughly provides insights into the fundamental roles of these three *RtHSFA9* genes in the heat stress response, which provides novel understandings regarding the thermal adaption ability of *R. tomentosa* and supports the future molecular breeding of thermotolerant trees.

## Data Availability

All the data are present in the manuscript will be available with this open-access publication.

## Supplementary Materials

The following supporting information can be downloaded at: https://www.mdpi.com/article/10.3390/life14121591/s1. Figure S1: The expression levels of *RtHSFA9s* in transgenic *Arabidopsis* plants, Table S1: List of primers used in this study.
