# Supplementary material for: RtHSFA9s of Rhodomyrtus tomentosa Positively Regulate Thermotolerance by Transcriptionally Activating RtHSFA2s and RtHSPs
Source: Life (Basel). 2024 Dec 2;14(12):1591. doi: 10.3390/life14121591 (PMC11676978; doi:10.3390/life14121591)
Supplement: Supplementary file 1 [file life-14-01591-s001.zip › Figure S1.pdf]

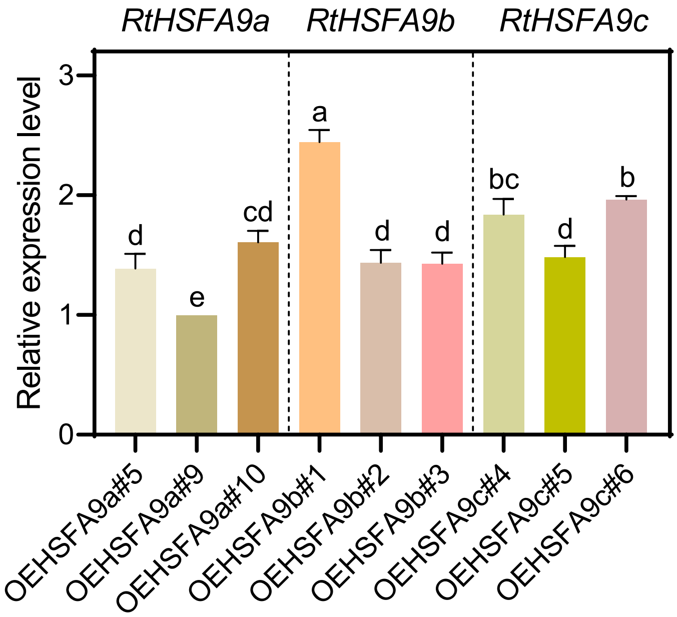


**Figure S1.** The relative mRNA abundance of *RtHSFA9s* in transgenic *Arabidopsis* plants. The relative expression levels of *RtHSFA9a*, *RtHSFA9b*, and *RtHSFA9c* in their overexpression lines were normalized to the line with the lowest expression (line OEHSFA9a#9). The *AtActin2* was used as the endogenous control. Data shown are means ± SD (n=3). The significances among different lines were evaluated by one-way ANOVA followed by post-hoc Tukey's HSD ($p < 0.05$). Samples sharing the same letters showed no significant difference.
